# CORN—Condition Orientated Regulatory Networks: bridging conditions to gene networks

**DOI:** 10.1093/bib/bbac402

**Published:** 2022-09-17

**Authors:** Ricky Wai Tak Leung, Xiaosen Jiang, Xueqing Zong, Yanhong Zhang, Xinlin Hu, Yaohua Hu, Jing Qin

**Affiliations:** School of Pharmaceutical Sciences (Shenzhen), Sun Yat-sen University, Shenzhen, 518107, China; College of Professional and Continuing Education, The Hong Kong Polytechnic University, Kowloon, Hong Kong, China; College of Life Sciences, University of Chinese Academy of Sciences, Beijing 100049, China; School of Pharmaceutical Sciences (Shenzhen), Sun Yat-sen University, Shenzhen, 518107, China; School of Pharmaceutical Sciences (Shenzhen), Sun Yat-sen University, Shenzhen, 518107, China; College of Mathematics and Statistics, Shenzhen Key Laboratory of Advanced Machine Learning and Applications, Guangdong Key Laboratory of Intelligent Information Processing, Shenzhen University, Shenzhen 518060, China; Department of Applied Mathematics, The Hong Kong Polytechnic University, Kowloon, Hong Kong, China; College of Mathematics and Statistics, Shenzhen Key Laboratory of Advanced Machine Learning and Applications, Guangdong Key Laboratory of Intelligent Information Processing, Shenzhen University, Shenzhen 518060, China; School of Pharmaceutical Sciences (Shenzhen), Sun Yat-sen University, Shenzhen, 518107, China

**Keywords:** conditions, regulatory networks, small molecules, transcriptional control, drugs

## Abstract

A transcriptional regulatory network (TRN) is a collection of transcription regulators with their associated downstream genes, which is highly condition-specific. Understanding how cell states can be programmed through small molecules/drugs or conditions by modulating the whole gene expression system granted us the potential to amend abnormal cells and cure diseases. Condition Orientated Regulatory Networks (CORN, https://qinlab.sysu.edu.cn/home) is a library of condition (small molecule/drug treatments and gene knockdowns)-based transcriptional regulatory sub-networks (TRSNs) that come with an online TRSN matching tool. It allows users to browse condition-associated TRSNs or match those TRSNs by inputting transcriptomic changes of interest. CORN utilizes transcriptomic changes data after specific conditional treatment in cells, and *in vivo* transcription factor (TF) binding data in cells, by combining TF binding information and calculations of significant expression alterations of TFs and genes after the conditional treatments, TRNs under the effect of different conditions were constructed. In short, CORN associated 1805 different types of specific conditions (small molecule/drug treatments and gene knockdowns) to 9553 TRSNs in 25 human cell lines, involving 204TFs. By linking and curating specific conditions to responsive TRNs, the scientific community can now perceive how TRNs are altered and controlled by conditions alone in an organized manner for the first time. This study demonstrated with examples that CORN can aid the understanding of molecular pathology, pharmacology and drug repositioning, and screened drugs with high potential for cancer and coronavirus disease 2019 (COVID-19) treatments.

## Introduction

### The tradition and novel approaches of drug development and repositioning

Traditionally, the ‘one gene, one drug, one disease’ paradigm was the leading philosophy in the field of rational drug design [1, 2]. However, cell systems are composed of networks instead of linear pathways, where intrinsic buffering mechanisms against the effect of single-target drugs are present due to functional redundancy and compensatory routes [3]. New development of drugs had been saturated in some disciplines in the 1991–2000 [4]. Therefore, a new strategy has also been adopted in recent years by embracing the idea of multitarget therapy [5, 6]. Rather than targeting a single target in one pathway, drugs targeting multiple targets and modulating the whole system have been demonstrated to be more efficient with less toxicity and less prone to drug resistance in some specific cases [7–9]. A deeper understanding of disease and pharmacological mechanisms in terms of gene regulatory systems is able to aid drug repositioning. And such repositioning is especially important during times of emergency such as COVID-19 when time is running short for novel drug development and manufacturing. Severe Acute Respiratory Syndrome Coronavirus-2 (SARS-CoV-2)’s targets and their associated biological processes were associated into COVID-19 related biological networks. These networks were used for studying the disease pathology as well as the screening of potential drugs for COVID-19 treatment [10, 11].

### Transcriptional regulatory networks (TRNs) can be controlled through small molecules/drugs

Over the past decades, novel advancements in sequencing technology enable us to gather different types of biological information such as genome, transcriptome and protein to DNA interactome in a fast and economical way. With the knowledge of how transcriptions are regulated, the integration of the mentioned biological information can reveal the gene regulatory network from behind by defining regulatory interactions among TFs and their target genes, constructing TRNs. A TRN is a collection of all transcription regulators with their associated downstream gene edges, which is highly condition-specific. The TRNs activated in a cell define its cell identity and function, where abnormal cell states lead to pathological conditions. Small molecule and drug treatments were shown to alternate the expression of transcription regulators by ligand targeting to their interacting partners [12]. For example, vorinostat is a drug targeting and inhibiting histone deacetylases (HDACs) [13], while HDACs were found to interact with the transcription factor (TF) family of regulatory factor for X-box and alternate the expression of downstream gene [14]. In other words, small molecules are able to affect TRN, and TRN can in turn govern the overall gene expression system in a cell, thus programming cell states. Treating disease through manipulation of gene regulatory networks has already been discussed in the field of precision medicine [15].

### The need for a Condition Orientated Regulatory Network database

Understanding how cell states can be programmed through small molecules/drugs or conditions by modulating the whole gene expression system granted us the potential to amend abnormal cells and cure diseases by repositioning existing drugs and molecules. To visualize how small molecules target TRN and modulate the gene regulatory system as a whole, this study utilized Connectivity map (Cmap) data [12] and chromatin immunoprecipitation coupled with sequencing or microarray (ChIP-X) data from Cistrome [16], where Cmap profiled transcriptomic changes after specific conditional treatment in cells, and ChIP-X data documented TF bindings in cells. By combining TF binding information and calculations of significant expression alterations of TFs and genes after the conditional treatments, single TF-centered transcriptional regulatory sub-networks (TRSNs) were constructed for each differentially expressed TF under each condition. The TRSNs were utilized as the basal units and deposited in an online platform: Condition Orientated Regulatory Networks (CORN, https://qinlab.sysu.edu.cn/home).

## Results and discussion

### Platform overview

**Figure 1:**
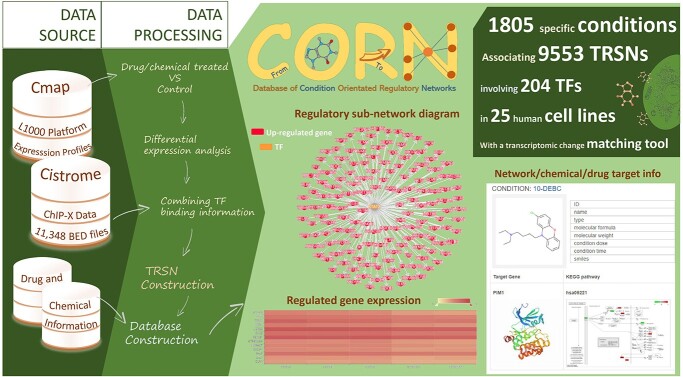
Overview of CORN including data source, data processing and features. Transcriptomic data from Cmap [12], ChIP-X data from Cistrome [16] and chemical information from various database were used as data sources for the construction of CORN. By combining TF binding information and calculations of significant expression alterations of TFs and genes after the conditional treatments, TRSNs under the effect of different conditions were constructed. See text for details.

CORN is an online platform of Condition Orientated Regulatory Networks. It is a library of condition (small molecule/drug treatments and gene knockdowns)-based TRSNs, users are able to search and browse interested TRN and TRSNs by inputting the concerning condition, cell line or TF. The resulting condition-associated TRSN page shows a vast amount of information including chemical information of the drug/molecule, the TF responsible for the regulation of the network, genes regulated, vector and fold change of such regulation as well as a TRSN diagram (Figure 1). Additional information such as previously annotated biological pathways of the TF and the drug target gene are also curated in the platform. Users can browse corresponding TRN freely by searching through conditions, cell lines, TFs or target genes that they are interested in. Additionally, our database also provides further useful information such as molecular structures and formula of the molecule used in the corresponding condition, protein structures of the curated target of the conditional molecule and the differential expressed genes in the corresponding TRSN. An online tool was also developed to enable users to identify the closest complimentary TRSNs with a set of input Differentially Expressed Genes (DEGs), and thus, the associated condition can be considered as a candidate to regulate or even cure a certain abnormal cell state (Supplementary Figure S1, see Supplementary Data available online at https://academic.oup.com/bib). A user guide with tutorial videos is available on the platform for the platform’s quick and easy use. TRNs were broken down into single TF-centered TRSNs as the basal unit of the platform instead of the whole TRN. As the regulatory relationship between a TF to its downstream genes is relatively more stable and consistent, they tend to be switched on and off together as a whole group. On the other hand, the complete sets of TRNs between different conditions are often partially altered due to the diverse expression changes of different TFs and are not turned on and off together as a whole network. Therefore, it will be more meaningful and useful to provide single TF-centered TRSNs as the basal unit of the platform. Moreover, when users search under a specific condition, the result page would show all the single TF-centered TRSNs (the collection of all transcription regulators with their associated downstream genes—TRN).

### Comparison over other platforms

**Figure 2:**
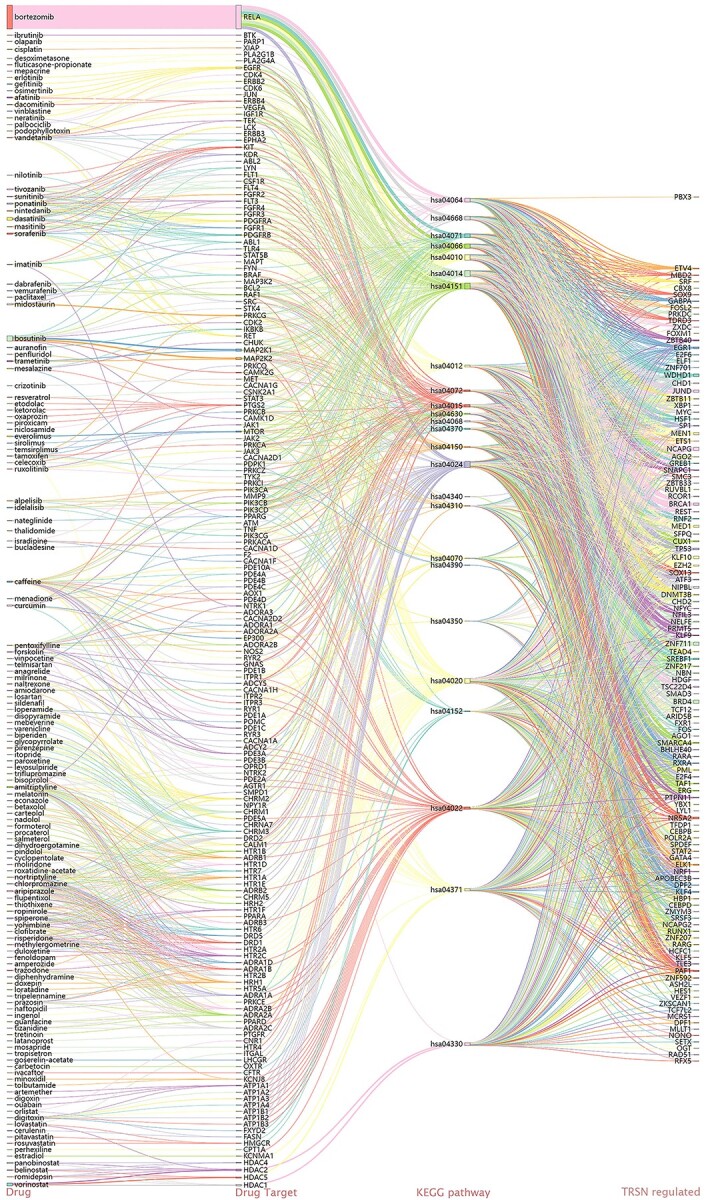
The connection between drugs, annotated drug target genes, annotated KEGG pathways involved and the TRSNs regulated computed by this study. A Sankey diagram that shows the connection between 141 launched drugs, 177 of their annotated target genes, 25 KEGG pathways that are involved with the target gene and the 117 TRSNs that are computed to be regulated by such drugs. The connection between previously annotated KEGG pathways and the TRSNs computed in this study are largely in unconformity. For drugs in clinical trials and preclinical phases, refer Supplementary Figures S2 and S3 (see Supplementary Data available online at https://academic.oup.com/bib).

CORN associated 1805 different types of specific conditions (small molecule/drug treatments and gene knockdowns) to 9553 TRSNs in 25 human cell lines, involving 204 TFs (Figure 1) by the integration of respective *in vivo* cell specific ChIP-X data and gene expression profile. Users can browse corresponding TRNs by searching through conditions, cell lines, TFs or target genes that they are interested in freely. This database is the first of its kind, with no similar database sharing the same function and usage. Database such as Cmap [12] is a transcriptome database recording transcriptomic regulatory network databases such as RegNetwork [17] integrate pre-discovered linear regulatory relationships between genes without the construction of regulatory networks by computations. GRNdb associated human and mouse TFs and downstream genes by TRN construction in various normal and pathological tissues utilizing inferred TF bindings [18], yet both databases do not focus on drugs/small molecules and genetic perturbations induced regulatory changes. Many of these drugs or small molecules were annotated to be associated with certain target genes and Kyoto Encyclopedia of Genes and Genomes (KEGG) pathways [19] before. KEGG mainly curates genomic information, chemical information and signaling pathway information directly without regulatory network constructions from literatures and various data source forming an enormous database. CORN is relatively smaller in terms of information recorded, yet it links drugs/small molecules and genetic perturbations to TF controlled gene regulatory networks by computational calculation based on *in vivo* TF binding information and gene expression data in respective cells. Moreover, pathway members in KEGG could be originated from different regulatory levels, which are not necessarily expressed simultaneously and thus not necessarily interacting with each other directly. On the other hand, gene members in a CORN TRSN are on the same regulatory level and controlled by the same TF at the same temporal state directly. In other words, the curated drugs/genes in CORN have direct control over gene members in the respective TRSNs under the same regulatory level, while members involved in the same KEGG pathway do not necessarily represent a direct controlling relationship over each other. Both KEGG pathway and CORN TRSN provide meaningful information concerning biological interactions, but they are orientated from different view angles. The regulatory relationships stated in CORN are more specific, while KEGG pathways are curated to be less explicit but in form of much larger networks, as dozens of CORN TRSNs can be seen to be joined under the same KEGG pathway (Figure 2). No doubt that the KEGG pathway database is extremely valuable to the scientific community; moreover, CORN will be equally valuable to provide key biological interaction information from another viewpoint which is more helpful for real time cell state manipulation.

### Validations

To verify whether the TRSN constructed can truly connect concerned drugs with regulated genes, we validated the replicability of our calculated association between small molecules and regulated genes by inputting publicly available drug induced transcriptomic change datasets from the Gene Expression Omnibus database (NCBI GEO) [20] in our matching tool, where the data were generated by different research groups yet still utilized the same cell line and drugs in their experiments. For example, NCBI GEO dataset GSE19638 recorded the transcriptomic changes induced by treatment of doxorubicin for 6 h in the cell line MCF7. The transcriptomic change was able to match with TRSN CN4305 (https://qinlab.sysu.edu.cn/home/report/CN4305) in our platform with the Forward-Backward greedy algorithm (FoBa) generated matching score of −0.214. The larger the number of the matching score represents a closer resemblance between the TRSN and the differential expression profile. A negative score represents the inputted differential expressed genes that were in the same regulatory direction with the reference TRSN. CN4305 was induced by the condition of 6 h daunorubicin treatment in MCF7 cells. While doxorubicin is just the 14-hydroxylated version of daunorubicin. Another three more sets of treatment-induced transcriptomic changes were validated by the matching tool (Supplementary Table S1, see Supplementary Data available online at https://academic.oup.com/bib). All matched the respective treatment drugs and cell lines utilized, proving that there are meaningful and consistent connections between small molecule treatments and the regulated genes in the TRSNs computed in our platform.

### Drugs capable to manipulate epithelial–mesenchymal transition through regulating TRNs

**Figure 3:**
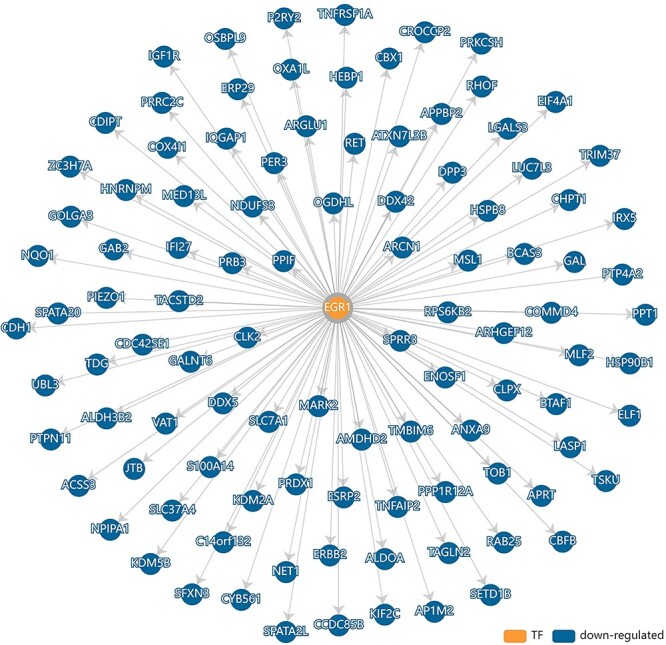
EGR1 TRSN induced by preclinical drug SAR245409. 103 genes are under the regulation of SAR245409 and EGR1, which are enriched with the GO molecular function annotation—‘cadherin binding’. For details, refer text and the TRSN report in CORN (https://qinlab.sysu.edu.cn/home/report/CN7356).

The epithelial–mesenchymal transition (EMT) is a process by which epithelial cells lose cell–cell adhesion and gain migratory ability to transit into mesenchymal cells [21]. As it is a hallmark process in cancer progression, gaining the ability to control such cell state conversion means acquiring the power to suppress cancer. To examine whether there are any small molecules/drugs/conditions that possess the potential of rectifying EMT, three datasets of transcriptomic changes recorded during EMT downloaded from NCBI GEO [20] were subjected to the TRSN matching tool. The results are summarized in Table 1 with the information of EMT dataset GEO accession number, CORN network IDs of TRSN matched, drugs utilized and TFs associated. In dataset GSE58252, it tracked the gene expression profile during a SNAIL-induced epithelial-to-mesenchymal transition in breast cancer cell line MCF-7 [22]. The transcriptomic change matches with TRNs such as CN7356 and CN8833 in our database. In CN7356, this TRSN is regulated by the preclinical drug SAR245409 which is a potent PI3K/mTOR pathway inhibitor known to induce apoptosis [23], yet the detailed mechanism of how SAR245409 works is poorly understood. Here in our database, we have associated SAR245409’s activity with the TRSN controlled by TF EGR1 with 103 genes under its regulation (Figure 3). The 103 regulated genes were enriched with the GO molecular function—‘cadherin binding’ (Supplementary Table S2, see Supplementary Data available online at https://academic.oup.com/bib), which plays a key role in the cell-to-cell adhesion process [24], where EGR1 has been known to play a key role in degenerating cell-to-cell adhesion [25] and promote metastasis [26]. Therefore, SAR245409 can demote cancer progression by inhibiting EMT through EGR-controlled TRSN. Thus, a more detailed mechanism to explain the efficacy of the preclinical anti-cancer drug SAR245409 was demonstrated and provided through CORN, aiding the understanding of molecular pathology and pharmacology. For CN8833, the APOBEC3B TRN was associated with a launched drug idoxuridine. The APOBEC3B was known to be a DNA Cytosine Deaminase that causes DNA mutations and is the molecular driver for various human cancers [27–32]. Intriguingly, idoxuridine is a nucleoside analog used as one of the first antiviral drugs approved in 1963 [33]. It is possible that the nucleoside analog activity of idoxuridine can inhibit the DNA Cytosine Deaminase activity of APOBEC3B and has the potential to prevent cancer development. Here for the first time in the field, we would like to suggest the repositioning of idoxuridine from a conventional antiviral to a potential novel drug for cancer inhibition and prevention.

**Table 1:** Drugs and TRSNs that matched with EMT. EMT datasets from the GEO database matched with the TRSNs in our platform with positive score are listed. The information of respective GEO Accession number from GEO, Network ID from our platform, as well as the associated drug name, TF and matching score are listed

EMT dataset	TRSN matched	Drug	TF associated	Matching score
GSE58252	CN7356	SAR-245409	EGR1	1.196
GSE58252	CN8833	Idoxuridin	APOBEC3B	1.107
GSE58252	CN4212	Gemcitabine	KLF4	0.789
GSE58252	CN1612	BRD-K81813927	EZH2	0.777
GSE58252	CN6198	Mitoxantrone	ZBTB17	0.705
GSE58252	CN1062	Cerivastatin	TAF1	0.597
GSE58252	CN1063	Cerivastatin	TAF1	0.597
GSE101809	CN3523	PYGO1	JUND	0.044
GSE101809	CN7916	AZ-20	TP53	0.038
GSE101809	CN5348	Tretinoin	TLE3	0.033
GSE101809	CN11	Phorbol-myristate-acetate	CEBPB	0.019
GSE136780	CN371	CT-200783	FOS	0.350
GSE136780	CN6477	GSK-2126458	TCF7L2	0.344
GSE136780	CN929	Lovastatin	JUND	0.268

### Potential drugs for COVID-19 treatment

The outbreak of coronavirus disease 2019 (COVID-19) has caused millions of deaths and suffering worldwide, and our global society is still in lack of effective treatment strategies to cope with the pandemic [34]. To screen drugs that have the potential for COVID-19 treatment, we have retrieved and compared the transcriptomic profiles between healthy individuals and patients with severe COVID-19 (GSE164805). All genes and their Log2 fold changes without any pre-set threshold were input into the CORN matching tool, TRNs with conditions associated with trichostatin-a (TSA) and BRD-K85133207 (HDAC1-selective) were matched (Supplementary Table S3, see Supplementary Data available online at https://academic.oup.com/bib), for which both conditions are concerned with HDAC inhibition. It is believed that upon the interaction between the viral spike protein and the human cell surface receptor ACE2, the HDAC pathway will be modulated while the HDAC pathway plays an important role in the pathogenicity of SRAS-CoV-2 and is the putative target for existing anti-COVID-19 therapeutics [35]. Indeed, TSA has been found to dock with the catalytic site of the SARS-CoV-2 main protease with good complementarity and inhibit the activity and replication of the virus *in vitro* [36]. Confirming the practicality and functionality of the CORN matching tool, while suggesting TSA and BRD-K85133207 to be novel COVID-19 drug candidates.

## Methods

### Database implementation

CORN implements a Linux-Apache-MySQL-PHP (LAMP) system. Data were saved in MySQL database. The web is constructed based on the powerful PHP framework CodeIgniter, which provides an Application Programming Interface (API) to connect the web to MySQL database. JavaScript libraries including jQuery (2.2.0), jQuery-labelauty and additional visualization plugin-ECharts were used to perform dynamic web services.

### Data retrieval

A total of 11 348 BED files associated with human TF ChIP-X data were downloaded from Cistrome Data Browser [16]. Transcriptomic data originated from 25 200 interference methods (19 811 small molecule compounds, 314 biological products, shRNA or cDNA, etc.) targeting 5075 target genes in human cell lines were downloaded from the Cmap project [12]. Chemical and physical information of different drugs and molecules were downloaded from The Drug Repurposing Hub [37], BD2K-LINCS Data Coordination and Integration Center [38], LINCS data portal [39], PubChem [40], ZINC [41], HMS LINCS [42] and CHEMBL [43] (Supplementary Table S4, see Supplementary Data available online at https://academic.oup.com/bib. Biological pathways associated with the drug target gene and the TF were retrieved from KEGG [19]. Transcriptomic data for validations and drug screening was downloaded from NCBI GEO [20].

### Data processing

After matching the transcriptomic data and ChIP-X data, 25 human cell lines were found to be included in both types of data. Thus, data originated from these cell lines were utilized for further data processing. ChIP-X data from the same cell line were merged and classified under the same BED file, while overlapping regions in a BED file were merged. Then, the merged regions in BED files were sorted according to their positions at chromosomes they originated from. An R package Limma [44] was used to conduct differential expression analyses on the transcriptomic data from Cmap to obtain Log2-transformed expression changes and adjusted *P*-values for each gene under different conditional treatment (using functions of lmfit and eBayes). Genes were considered differentially expressed with the absolute Log2 fold change value greater than 0.5 and the adjusted *P*-value less than 0.01. GO enrichment test was done by Protein Analysis Through Evolutionary Relationships Overrepresentation Test with default settings using Fisher’s exact test and the threshold of Benjamini–Hochberg False Discovery Rate correction was set to 0.05 [45].

### TRSN constructions

Significant differential expressed data resulting from Limma (absolute value of Log2 fold change >0.5 and adjusted *P*-value <0.01) were selected to perform the following processes. ChIP-X and transcriptomic data from the same cell line were then paired and subsequent to target analysis by integration of both data with BETA [46]. Log2-transformed expression changes and adjusted *P*-values of all genes reported by Limma were parsed into BETA compatible format. Condition-specific TRNs were constructed using BETA 1.0.7 with each pair of ChIP-X and transcriptome data, linking each differential expressed TF to their respective 200 most significant up-regulated and 200 most down-regulated genes under each specific condition into two TRSNs (one set of up-regulated genes containing TRSN, another set of TRSN with down-regulated genes). In this process, the parameters of BETA were set as basic, −k LIM, −da 200 utilizing genome information from *Homo sapiens* (human) genome assembly GRCh38 (hg38). As a result, a total number of 9553 condition-specific TRSNs were generated.

### Matching tool construction

9553 TRNs were then merged into one single matrix *A*. Among these 9553 TRSNs, 114 TRSNs were paired from different regulatory directions (up-regulated and down-regulated) of the same condition-specific TF, which were then merged into 57 columns. Afterwards, they were grouped with 9439 single regulatory direction TRSNs, these TRSNs form a matrix *A* with 10 061 rows (genes) and 9496 columns (condition-specific TRSNs). Each column represents a TRSN *j* controlled by one TF *k* under a certain condition *l*, and each row is a gene *i* that probably regulated by those TFs. If gene *i* is regulated by TF *k* under condition *l*, *A_ij_* is the expression Log2 fold change of gene *i* under condition *l*, otherwise, *A_ij_* is equal to 0. Expression data inputted by users form matrix *B.* It can be any transcriptomic changes (Log2 fold changes), such as comparison between patients and corresponding control individuals, but it must only contain two columns, Symbol and LogFC.

To identify TRNs that best match transcriptome changes inputted by users, a sparse learning model was applied based on the assumption that a small number of the condition-specific TRSNs will be selected if they are dysregulated in the transcriptomic changes inputted by the users. In particular, *b* is the data vector in matrix *B*, and *x* is the coefficient vector representing the association between TRSNs and the inputted transcriptomic changes. Then, the sparse learning model for inferring its perturbed TRNs can be formulated by

$${\displaystyle \begin{array}{cc}\min & {\Big\Vert Ax-b\Big\Vert}^2\\{}\textrm{s}.\textrm{t}.& \operatorname{card}(x)\le s,\end{array}}$$ where card(*x*) denotes the cardinality (i.e. the number of nonzero coefficients) of variable vector *x*, and *s* is the given sparsity that represents the number of selected TRSNs. *s* was arbitrarily set as 10 in this study to search up to 10 most correlated TRSNs for each input, with the generation of respective matching score. Adaptive FoBa was used to find best matched TRSNs, as the traditional ordinary least squares method does not pay much attention to the sparsity of the results in the optimization process, while FoBa possesses superior performance and computational feasibility over other greedy algorithms [47]. The matching score is used to reflect the contribution of different TRSNs fitting into the inputted *b*. In short, the larger the magnitude of the resulted score would represent a closer resemblance between the TRSN and the differential expression profile. A negative score would represent the regulatory direction of the inputted differential expressed genes that were in tune with the regulatory direction of the reference TRSN. If the readers possess a set of DEGs induced by an unknown cause, exploring matching TRSNs with negative scores would be useful for these readers. It is because the transcriptional controller/condition of the negatively scored TRN could cause a similar expression profile change in the same regulatory direction as their inputted DEGs (see ‘Validations’ in Results and Discussions), which means they might also be the potential causes of such inputted differential expression. As for positive scores, they represent the inputted DEGs were in the counter regulatory direction to the matched TRSN. Searching condition oriented TRSNs with positive scores would be helpful for readers who are seeking conditions/drugs with the therapeutic potential for counteracting the expression changes induced by a pathological state (see ‘Potential drugs for COVID-19 treatment’ and ‘Drugs capable to manipulate EMT through regulating TRNs’ in Results and Discussions).

## Conclusion

The online platform of condition-associated regulatory networks, CORN curated 9553 condition triggered transcription regulatory sub-networks, associated to 1805 specific conditions, 204 TFs and 25 human cell lines. Researchers can search and browse TRNs inferred from experimental results by selecting through different interested conditions. By linking and curating specific conditions to the responsive TRSNs, CORN enables the scientific community to perceive how TRSNs are altered and controlled by conditions in an organized manner for the first time while gain more knowledge about abnormal cell states and diseases. Utilizing the transcriptional change matching tool from CORN, several drugs were found to be potentially repositioned for appending pathological cell states such as cancer and COVID-19. For example, SAR245409 is possible to demote cancer progression by inhibiting EMT through EGR-controlled TRSN, while idoxuridine is likely to inhibit the DNA Cytosine Deaminase activity of APOBEC3B and has the potential to prevent cancer development. TSA and BRD-K85133207 have the potential to modulate the HDAC pathway during SRAS-CoV-2 infection, reducing the pathogenicity of COVID-19. This platform can be the foundation and reference material for scientists who aim to control TRNs and cell states with small molecules or manipulation of various conditions. It provides key resources for the scientific community to study the relationships between conditions and cell states in terms of gene regulations. By demonstrating with examples how various conditions can regulate TRN, our database has a wide range of contribution ranging from execution of condition-based cell type conversion to appending pathological cell states.

## Authors’ contributions

R.W.T.L. interpreted the data, drafted and revised the manuscript. X.J. built the online platform. X.Z. and Y.Z. analyzed the data. X.H. built the matching tool. Y.H. and J.Q. contributed to the study design and manuscript revision.

## Data availability

The data and codes underlying this article are available at https://qinlab.sysu.edu.cn/home

**Key Points**

- This study associated 1805 different types of specific conditions (small molecule/drug treatments and gene knockdowns) to 9553 TRSNs in 25 human cell lines, involving 204 TFs.
- An online TRSN matching tool was developed allowing users to browse condition-associated TRSNs or match those TRSNs by inputting transcriptomic changes of interest.
- This platform can aid the understanding of molecular pathology, pharmacology and drug repositioning and have demonstrated the drug screenings for cancer and COVID-19 treatment with our platform.

## Supplementary Material

Supplementary_information_bbac402

Table_S4_bbac402

## Author Biographies

**Ricky Wai Tak Leung** is a Lecturer at the College of Professional and Continuing Education, The Hong Kong Polytechnic University. His research focuses on unraveling important biological phenomena with bioinformatics. Understanding functional, pharmaceutical, and pathological conditions in animals as well as evolutionary relationships among animals and viruses via multi-omics data analyses.

**Xiaosen Jiang** is a postgraduate student at the College of Life Sciences, University of Chinese Academy of Sciences. His research focuses on bioinformatics analyzes and gene regulatory network constructions.

**Xueqing Zong** is a postgraduate student at the School of Pharmaceutical Sciences (Shenzhen), Sun Yat-sen University. Her research focuses on small molecules to gene regulatory network relationships as well as their implication for disease therapeutics.

**Yanhong Zhang** is a postgraduate student at the School of Pharmaceutical Sciences (Shenzhen), Sun Yat-sen University. Her research focuses on conditions to gene regulatory network relationships.

**Xinlin Hu** is a postgraduate student at the Department of Applied Mathematics, The Hong Kong Polytechnic University. His research focuses on theory and algorithms for large-scale optimization in bioinformatics.

**Yaohua Hu** is an associate professor at the College of Mathematics and Statistics, Shenzhen University. His research focuses mainly on theory and algorithms for large-scale optimization, and their various applications in statistics, machine learning and bioinformatics.

**Jing Qin** is an associate professor at the School of Pharmaceutical Sciences (Shenzhen), Sun Yat-sen University. Her research focuses mainly on integrative OMICs approaches for gene regulation studies.
