# Supplementary material for: CORN—Condition Orientated Regulatory Networks: bridging conditions to gene networks
Source: Brief Bioinform. 2022 Sep 17;23(6):bbac402. doi: 10.1093/bib/bbac402 (PMC9677472; doi:10.1093/bib/bbac402)
Supplement: Supplementary_information_bbac402 [file supplementary_information_bbac402.docx]

**Supplementary information**

**CORN - Condition Orientated Regulatory Networks: bridging conditions to gene networks**

Ricky Wai Tak Leung^1,2,^^, Xiaosen Jiang^3,^^, Xueqing Zong^1,^^, Yanhong Zhang^1^, Xinlin Hu^4,5^, Yaohua Hu^4,*^, Jing Qin^1, *^

^1^ School of Pharmaceutical Sciences (Shenzhen), Sun Yat-sen University, Shenzhen, 518107, China

^2^ College of Professional and Continuing Education, The Hong Kong Polytechnic University, Kowloon, Hong Kong, China

^3^ College of Life Sciences, University of Chinese Academy of Sciences, Beijing 100049, China.

^4^ College of Mathematics and Statistics, Shenzhen Key Laboratory of Advanced Machine Learning and Applications, Guangdong Key Laboratory of Intelligent Information Processing, Shenzhen University, Shenzhen 518060, China.

^5^ Department of Applied Mathematics, The Hong Kong Polytechnic University, Kowloon, Hong Kong, China

^Equal contribution. *Corresponding author.

**Summary**

Biological systems are composed of complex and inter-connected gene regulatory networks instead of simple linear on and off regulatory pathways. A transcriptional regulatory network (TRN) is a collection of transcription regulators with their associated downstream genes, which is highly condition-specific. The TRNs activated in a cell define its cell identity and function, where abnormal cell states lead to pathological conditions. Small molecule and drug treatments were shown to alternate expressions of transcription regulators by ligand targeting. In other words, small molecules affect TRNs, and TRNs govern the overall gene expression system in a cell, thus programming cell states. Understanding how cell states can be programmed through small molecules/drugs or conditions by modulating the whole gene expression system granted us the potential to amend abnormal cells and cure diseases. To visualize how small molecules target TRN and modulate the whole gene regulatory system, we have constructed the Database of Condition Orientated Regulatory Networks (CORN, <https://qinlab.sysu.edu.cn/home>). This file contains the supplementary data of the manuscript, please refer to the following table of content for a specific supplementary figure or table that you are looking for.

**Tabe of contents**

| **Figure/Table** | **Page number** |
| --- | --- |
| Figure S1. Matching tool construction. | 3 |
| Figure S2. The connection between preclinical drugs, annotated drug target genes, annotated KEGG pathways involved, and the TRSNs regulated computed by this study. | 4-5 |
| Figure S3. The connection between drugs in clinical trials, annotated drug target genes, annotated KEGG pathways involved, and the TRSNs regulated computed by this study. | 6-7 |
| Table S1. Results of matching tool validation. | 8 |
| Table S2. GO analysis of the 103 genes controlled by transcription factors EGR1. | 9 |
| Table S3. The TRSNs matched to GSE164805. | 10 |
| Table S4. Records of Cmap IDs、 CORN network information、Cistrome IDs and sources of molecular information used in this study | In a separate excel file |


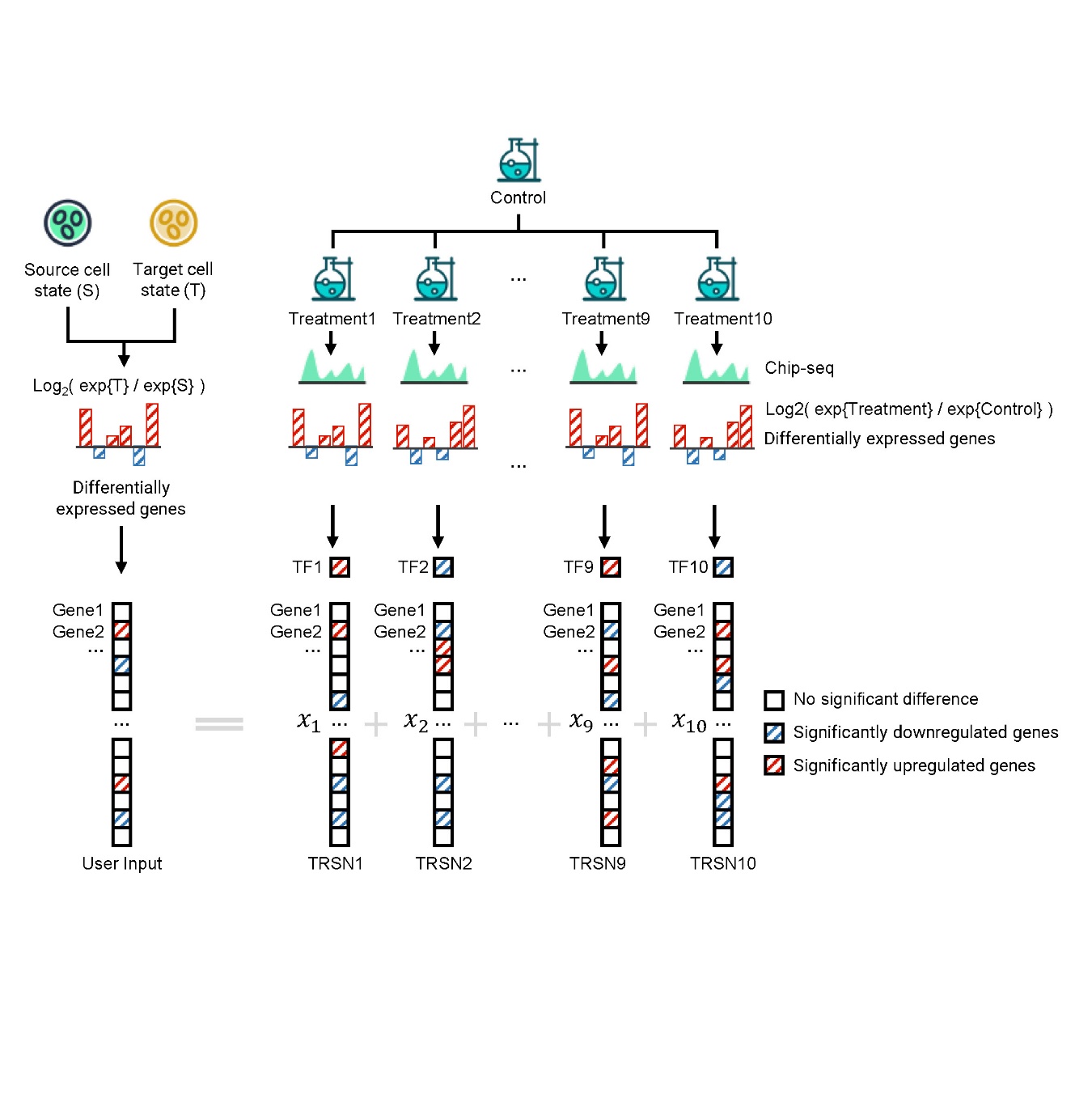


**Figure S1. Matching tool construction.** When users try to search for a condition that can reverse a change of cell state (for instance, make cancer cells normal again), they can input the expression changes (Log2 fold change) of differentially expressed genes. Our matching tool will match the 10 most correlated TRSNs from various treatments (eg. small molecules and gene manipulations) with matching scores. A negative score would represent the regulatory direction of the inputted differential expressed genes were in tune with the regulatory direction of the reference TRSN. As for positive scores, they represent the inputted DEGs were in the counter regulatory direction to the matched TRSN.


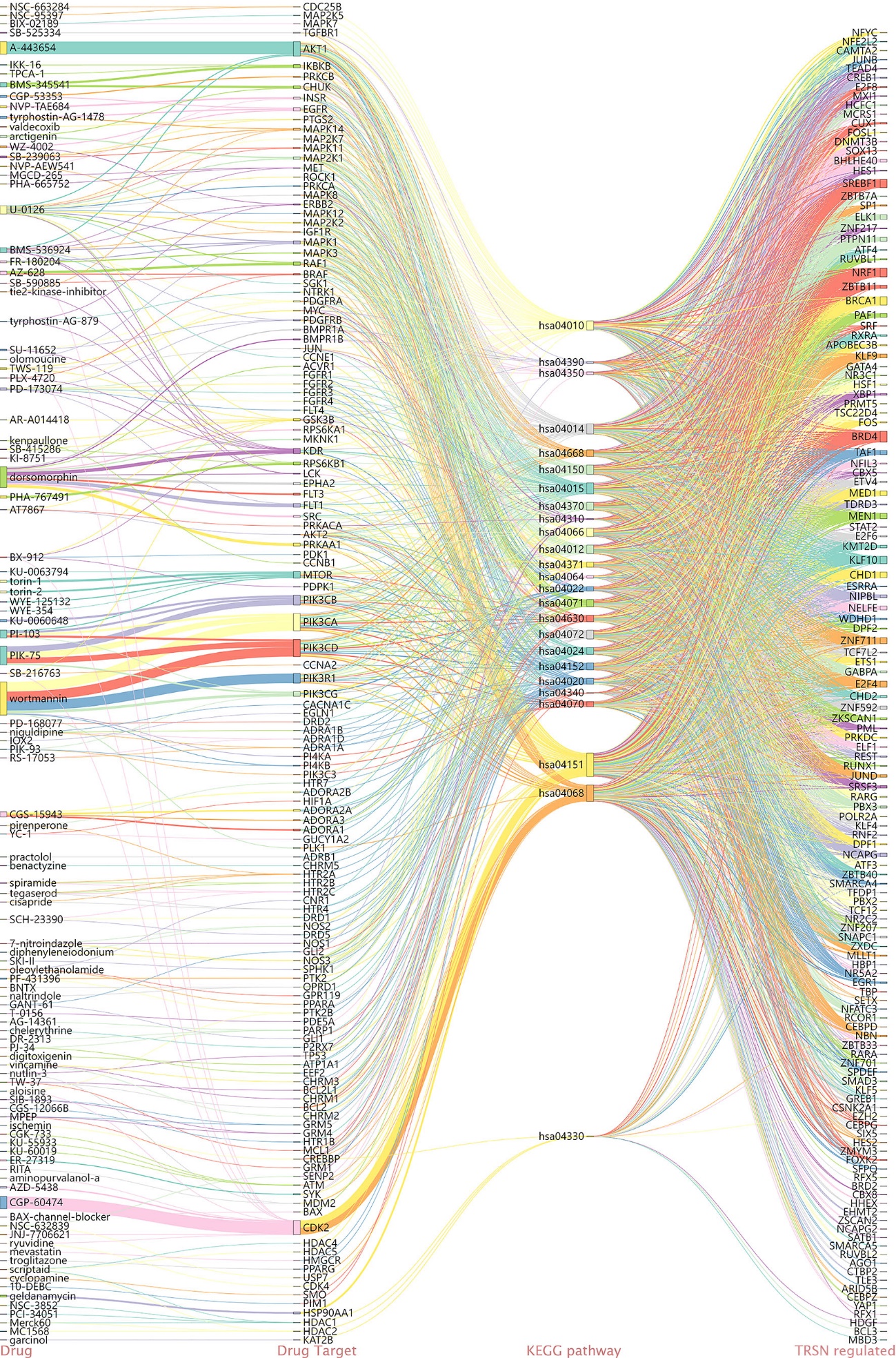


**Figure S2. The connection between preclinical drugs, annotated drug target genes, annotated KEGG pathways involved, and the TRSNs regulated computed by this study.** A Sankey diagram that shows the connection between 107 drugs in preclinical stage, 137 of their annotated target genes, 25 KEGG pathways that are involved with the target gene, and the 134 TRSNs that are computed to be regulated by such drugs.


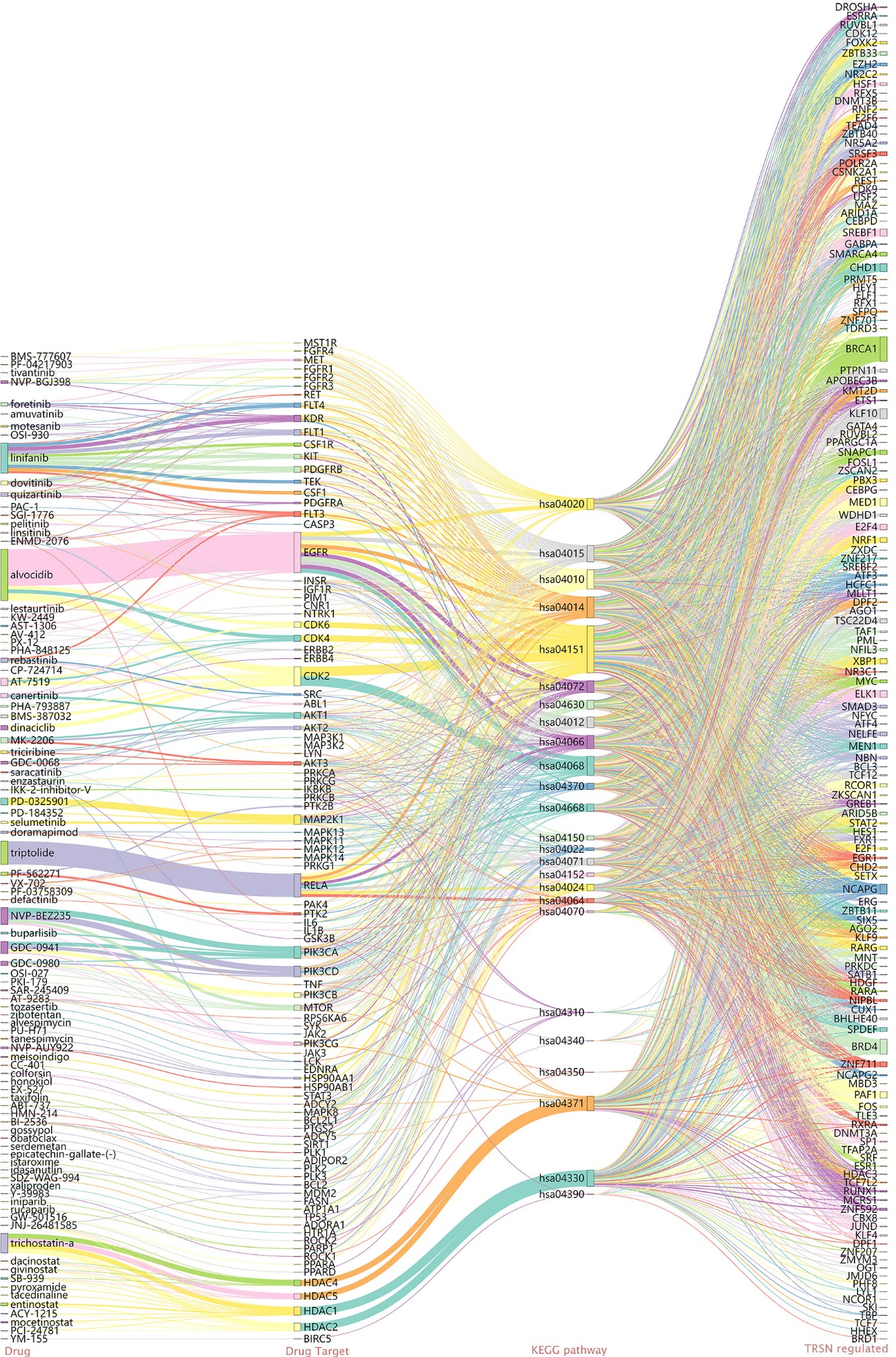


**Figure S3. The connection between drugs in clinical trials, annotated drug target genes, annotated KEGG pathways involved, and the TRSNs regulated computed by this study.** A Sankey diagram that shows the connection between 92 drugs in clinical trial, 96 of their annotated target genes, 25 KEGG pathways that are involved with the target gene, and the 139 TRSNs that are computed to be regulated by such drugs.

| **NCBI GEO dataset** | | | **TRSN matched in CORN** | | | |
| --- | --- | --- | --- | --- | --- | --- |
| **ID** | **treatment** | **cell** | **ID** | **matching score** | **treatment** | **cell** |
| GSE19638 | doxorubicin | MCF7 | CN4305 | -0.214 | doxorubicin | MCF7 |
| GSE19638 | SN38 | MCF7 | CN341 | -0.182 | SN38 | MCF7 |
| GSE16760 | triptolide | A549 | CN3965 | -0.166 | triptolide | A549 |
| GSE74572 | trichostatin | A549 | CN1433 | -0.126 | trichostatin | A549 |

**Table S1. Results of matching tool validation.** Drug-induced transcriptome changes downloaded from NCBI GEO, these datasets can be matched to the corresponding drug and cell line found in our database. Proving there are meaningful and consistent connections between small molecule treatments and the regulated genes in the TRSNs computed in our platform.

| Analysis Type: | PANTHER Overrepresentation Test (Released 20210224) | | | | | | |
| --- | --- | --- | --- | --- | --- | --- | --- |
| Annotation Version and Release Date: | GO Ontology database DOI: 10.5281/zenodo.5228828 Released 2021-08-18 | | | | | | |
| Analyzed List: | upload_1 (*Homo sapiens*) | | | | | | |
| Reference List: | *Homo sapiens* (all genes in database) | | | | | | |
| Test Type: | FISHER | | | | | | |
| Correction: | FDR | | | | | | |
| GO molecular function complete | *Homo sapiens* - REFLIST (20595) | upload_1 (110) | upload_1 (expected) | upload_1 (over/under) | upload_1 (fold Enrichment) | upload_1 (raw P-value) | upload_1 (FDR) |
| cadherin binding (GO:0045296) | 323 | 10 | 1.73 | + | 5.8 | 1.14E-05 | 1.86E-02 |
| protein binding (GO:0005515) | 14349 | 99 | 76.64 | + | 1.29 | 5.97E-07 | 2.91E-03 |
| binding (GO:0005488) | 16519 | 105 | 88.23 | + | 1.19 | 9.58E-06 | 2.34E-02 |

**Table S2. GO analysis of the 103 genes controlled by transcription factors EGR1.** The GO molecular function ‘cadherin binding’ is significantly enriched.

| Network matched | Condition | TF associated | Score |
| --- | --- | --- | --- |
| CN3704 | HDAC1-selective  (BRD-K85133207) | ELK1 | 0.708 |
| CN5210 | palbociclib | MYC | 0.480 |
| CN3954 | trichostatin-a | BHLHE40 | 0.258 |
| CN4611 | DKFZP761P0423 | PHF8 | 0.162 |

**Table S3. The TRSNs matched to GSE164805 with positive scores.** Among these conditions, Trichostatin-a, BRD-K85133207 are HDAC inhibitors.
